# A Pre-Training Framework Based on Multi-Order Acoustic Simulation for Replay Voice Spoofing Detection

**DOI:** 10.3390/s23167280

**Published:** 2023-08-20

**Authors:** Changhwan Go, Nam In Park, Oc-Yeub Jeon, Chanjun Chun

**Affiliations:** 1 Department of Computer Engineering, Chosun University, Gwangju 61452, Republic of Korea; chgo@chosun.ac.kr; 2 Digital Analysis Division, National Forensic Service, Wonju 26460, Republic of Korea; naminpark@korea.kr (N.I.P.); yeubjeon@korea.kr (O.-Y.J.)

**Keywords:** voice spoofing, acoustic configuration, deep learning

## Abstract

Voice spoofing attempts to break into a specific automatic speaker verification (ASV) system by forging the user’s voice and can be used through methods such as text-to-speech (TTS), voice conversion (VC), and replay attacks. Recently, deep learning-based voice spoofing countermeasures have been developed. However, the problem with replay is that it is difficult to construct a large number of datasets because it requires a physical recording process. To overcome these problems, this study proposes a pre-training framework based on multi-order acoustic simulation for replay voice spoofing detection. Multi-order acoustic simulation utilizes existing clean signal and room impulse response (RIR) datasets to generate audios, which simulate the various acoustic configurations of the original and replayed audios. The acoustic configuration refers to factors such as the microphone type, reverberation, time delay, and noise that may occur between a speaker and microphone during the recording process. We assume that a deep learning model trained on an audio that simulates the various acoustic configurations of the original and replayed audios can classify the acoustic configurations of the original and replay audios well. To validate this, we performed pre-training to classify the audio generated by the multi-order acoustic simulation into three classes: clean signal, audio simulating the acoustic configuration of the original audio, and audio simulating the acoustic configuration of the replay audio. We also set the weights of the pre-training model to the initial weights of the replay voice spoofing detection model using the existing replay voice spoofing dataset and then performed fine-tuning. To validate the effectiveness of the proposed method, we evaluated the performance of the conventional method without pre-training and proposed method using an objective metric, i.e., the accuracy and F1-score. As a result, the conventional method achieved an accuracy of 92.94%, F1-score of 86.92% and the proposed method achieved an accuracy of 98.16%, F1-score of 95.08%.

## 1. Introduction

Voice spoofing is the act of someone trying to break into a specific ASV system by forging the user’s voice. Recent advances in deep learning and hardware have made it possible for voice spoofing to evade the security of ASV systems. Representative voice spoofing techniques include the TTS, which converts text to audio, and VC, which converts someone else’s voice. Another method utilizes commercially available voice editing softwares to record the audio after sophisticated editing [1]. Voice spoofing can destroy the security of ASV systems. Therefore, it is necessary to develop countermeasures.

Conventional voice spoofing detection approaches include machine learning-based methods, such as the Gaussian mixture model (GMM) and support vector machine (SVM) [2,3], and deep learning-based methods, such as convolutional neural networks (CNN) and recurrent neural networks (RNN) [4,5]. As a representative example, there is a method that utilizes the deep learning model, such as the light convolutional neural network (LCNN) [6], using feature extraction techniques, such as the constant Q-transform cepstral coefficients (CQCC) and linear frequency cepstral coefficients (LFCC), to predict whether an input audio is bonafide or spoof [7,8,9,10,11]. In addition, interest in voice spoofing is growing, such as in the ASVspoof challenge [12], which is an international competition to detect voice spoofing. This challenge aims to detect two voice spoofing scenarios: a logical access (LA) task to detect voice spoofing through TTS or VC, and physical access (PA) task to detect replay voice spoofing. ASVspoof provides datasets for detecting the LA and PA. However, unlike the LA, which can generate voices, such as the TTS or VC through deep learning models, PA requires consideration of all the physical processes, such as the recording devices, speakers, and room paths. Therefore, it can be relatively difficult to construct a large dataset. In these problems, the ASVspoof2019 PA dataset consists of the original and replayed audio that considers only specific conditions. Specifically, it consists of 27 room acoustics: room size, RT60, and the distance between the user and microphone, which are divided into three categories: a, b, c, and nine replay configurations: distance of the user and speaker and recording device quality, which is divided into three categories: A, B, C [13].

Recently, a replay voice spoofing detection method that utilizes the acoustic configuration of the original and replay audio has been proposed with various advances, such as feature extraction techniques, constructing datasets, or utilizing the large existing datasets [14]. Here, acoustic configuration refers to the factors, which may occur during the recording process, such as the microphone type, reverberation, time delay, and noise between the speaker and recording device. Gupta et al. proposed a feature extraction technique to estimate the frequency distortion for replay voice spoofing detection and compared its performance with conventional feature extraction techniques [15]. However, these feature extraction techniques have the disadvantage of only considering specific acoustic configurations, such as frequency distortion. Baymann et al. proposed a replay voice spoofing detection method by constructing a dataset through a physical recording process using various recording devices and speakers in 10 different locations, including a car, classroom, kitchen, and bedroom [16]. These approaches have the advantage of considering various acoustic configurations. However, their disadvantage is that they do not solve the problem of constructing replay datasets. Another approach is transfer learning, which utilizes large datasets and is a popular technique in deep learning. A typical approach is to set the initial weights of a model pre-trained on a large dataset, such as the ImageNet, and performs fine-tuning it with the dataset for a specific task [17]. In general, the performance of a deep learning model increases with the amount of training data. The advantage of a model trained on a large amount of data is that it can generalize well to unseen data because it learns the general features of the data, thus mitigating over-fitting [18]. Shim et al. proposed a replay voice spoofing detection framework using self-supervised pre-training of acoustic configurations utilizing the voxceleb dataset [19], which comprises large-scale speaker recognition data built through YouTube sources [20]. This approach assumes that segments within the same utterance have the same acoustic configuration, perform pre-training to determine whether a pair of segments has the identical or different acoustic configuration, and then perform fine-tuning when training the replay voice spoofing detection model. However, because the voxceleb dataset consists only of the original audio clips, it can only consider the acoustic configuration of the original audio and not the acoustic configuration of the replay audio.

To overcome these limitations, this study proposes a pre-training framework based on multi-order acoustic simulation for replay voice spoofing detection. Multi-order acoustic simulation utilizes existing datasets of clean signals and RIRs to generate an audio that simulates different acoustic configurations of the original and replayed audio. We define a clean signal as the audio recorded with a high-quality microphone in a non-reverberant environment, such as a studio, and nth-order audio as the audio that has undergone *n* times a physical recording process that considers speakers, microphones, and room paths for the clean signal. In this case, we assume that the original audio corresponds to the 1st-order audio, which performs a single recording process, and that the replay audio corresponds to the 2nd-order audio. Since the audio may have acoustic configurations, such as the microphone type, reverberation, time delay, and noise during recording, we also assume that the 1st-order audio has one acoustic configuration and 2nd-order audio has two. In this study, we perform convolution with a clean signal and RIR to generate audio that simulates the acoustic configuration of 1st-order and 2nd-order audio. Signal convolution is the process of combining two signals to create a new signal, and multi-order acoustic simulation creates a new audio with an acoustic configuration by convolving the temporal characteristics, such as the frequency, amplitude, and phase of the clean signal, and spatial characteristics, such as the acoustic configuration of the RIR. Specifically, when simulating a 1st-order audio, we perform convolution of the clean signal and one RIR, and 2nd-order audio convolves two RIRs. We also assume that a model pre-trained on audio simulating different acoustic configuration of the 1st-order and 2nd-order audio generated by multi-order acoustic simulation can effectively classify different acoustic configurations of the original and replay audio. The overall framework of the proposed method involves performing a pre-training process to classify the audio generated by the multi-order acoustic simulation using the VCTK Corpus dataset [21] and Aachen impulse response dataset [22] into three classes: clean, 1st-order, and 2nd-order, and then performing fine-tuning when training the replay voice spoofing detection model using the ASVspoof2019 PA dataset. To demonstrate the effectiveness of the proposed method, we compared the performance of the proposed method with that of a conventional method that does not utilize pre-training through an objective evaluation metric, i.e., the accuracy. This paper is organized as follows: in Section 2, Section 3 and Section 4, we describe the definition of multi-order acoustic simulation and the overall framework of the proposed method, and in Section 5 and Section 6, we compare the performance of the proposed method with that of the conventional method without pre-training through an objective evaluation metric.

## 2. Multi-Order Acoustic Simulation

**Figure 1:**
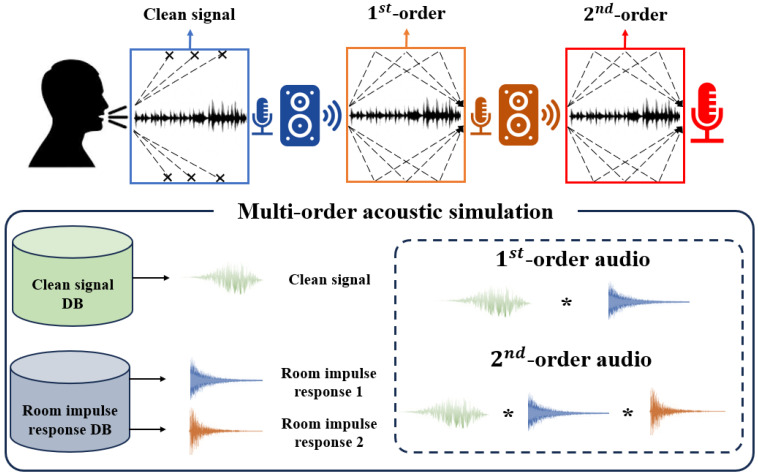
Definition of multi-order acoustic simulation for replay voice spoofing detection.

Figure 1 shows a multi-order acoustic simulation for replay voice spoofing detection. In this study, we assume that a clean signal is audio recorded in a non-reverberant environment, such as a studio. Additionallyy, we assume the original audio corresponds to the 1st-order audio, which performs one recording process considering the speaker, room path, and microphone for the clean signal, and the replay audio corresponds to the 2nd-order audio, which performs two recording processes. In addition, because the audio may have acoustic configurations during recording, we assume that the 1st-order audio has one acoustic configuration and 2nd-order audio has two. Multi-order acoustic simulation utilizes the existing clean signal and RIR dataset to generate the audio that simulates the acoustic configuration of the 1st-order and 2nd-order audios. When simulating the 1st-order audio, the clean signal and one RIR are convolved, and the 2nd-order audio is convolved with two RIRs. In addition, when the audio simulating the 1st-order audio is called $R_{1}$, and audio simulating the 2nd-order is called $R_{2}$, $R_{1}$ and $R_{2}$, using a clean signal and RIR, can be represented as:

(1) $$R_{1}\left(n\right)=s\left(n\right)*h_{1}\left(n\right)=\sum_{k=0}^{n-1}s\left(k\right)\cdot h_{1}\left(n-k\right)$$

(2) $$R_{2}\left(n\right)=R_{1}\left(n\right)*h_{2}\left(n\right)=\sum_{k=0}^{n-1}R_{1}\left(k\right)\cdot h_{2}\left(n-k\right)$$

where *n* is the index of the signal, *s* is the clean signal, and $h_{1}$ and $h_{2}$ are the different RIRs. Equation (1) shows the expression to generate $R_{1}$ by convolving the temporal characteristics, such as frequency, phase, and amplitude of *s*, and acoustic configurations, such as the microphone type, sound reduction, reverberation, and noise of $h_{1}$. Equation (2) shows the expression to generate $R_{2}$ by convolving the temporal characteristics of $R_{1}$ and acoustic configuration of $h_{2}$. The convolution of clean signals and RIR to generate the audio with an acoustic configuration has been utilized in various applications [23]. Research is being conducted to generate the RIR using techniques, such as the image method and fast-RIR, to simulate room acoustics in various environments without restrictions [24,25]. These RIR generation techniques can easily generate impulse responses considering the room size, sound reduction, time delay, reverberation, etc., and show high performance in simulating room acoustics [26]. However, the RIR generated by this technique may not be suitable for simulating the original and replay audio because it does not consider factors such as the non-linearity or distortion caused by the microphone.

Considering these problems, this study used the RIR datasets acquired using smartphones, which are the most accessible recording devices among the existing RIR datasets. Smartphones are rapidly evolving in hardware, and the performance of their built-in microphones is improving. Therefore, the threat of replay voice spoofing from smartphones may increase. Considering that, we used the Aachen impulse response dataset, which acquires the RIRs through a physical recording process using a smartphone. The Aachen impulse response dataset provides 214 RIRs that reproduce the situation of a user talking or listening to a meeting or lecture in various places, such as offices, kitchens, corridors, stairways, lecture rooms, and meeting rooms, using HEAD acoustics HMS II.3 artificial head and omnidirectional Beyerdynamic MM1 measurement microphones. In addition, we assumed the VCTK Corpus dataset to be a clean signal because the ASVspoof2019 PA dataset was created based on the VCTK Corpus dataset.

## 3. Replay Voice Spoofing Detection Framework

**Figure 2:**
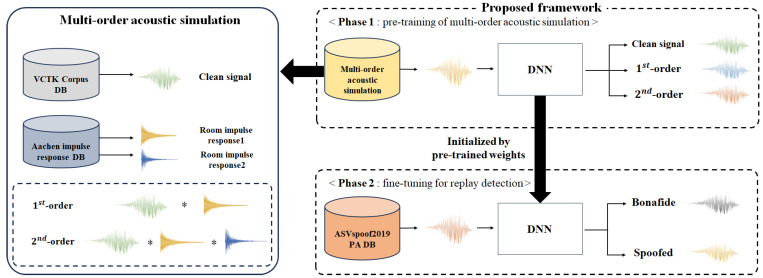
Multi-order acoustic simulation-based pre-training framework for replay voice spoofing detection.

Figure 2 shows the overall framework of the proposed method. Phase 1 of the proposed method is the pre-training process, which utilizes a multi-order acoustic simulation to classify three classes: clean signal, 1st-order, and 2nd-order. In the multi-order acoustic simulation, one clean signal and two different RIRs are randomly extracted from the VCTK Corpus and Aachen impulse response datasets to simulate the 1st-order and 2nd-order audio through convolution. The generated audio is used as the input to the pre-training model, which is trained to predict one of the following classes: clean signal, 1st-order, and 2nd-order. At this time, the input audio may be a clean signal or 1st-order and 2nd-order audio generated through a multi-order acoustic simulation. Phase 2 sets the weights of the pre-training model as the initial weights for training the replay voice spoofing detection model and then performs fine-tuning. The dataset used for replay voice spoofing detection is the ASVspoof2019 PA dataset, which predicts whether the input audio is bonafide or spoofed. In the proposed method, we assume that a pre-training model that utilizes multi-order acoustic simulation to classify the three classes, i.e., the clean, 1st-order, and 2nd-order, will be able to effectively classify the different acoustic configurations of the original and replay audio. In addition, we expect that the deep learning model can be generalized to unseen replay audio to some extent through the process of fine-tuning with ASVspoof2019 by utilizing the weights of the deep learning model that has learned the acoustic configuration of a large amount of the 1st-order and 2nd-order audio.

**Figure 3:**
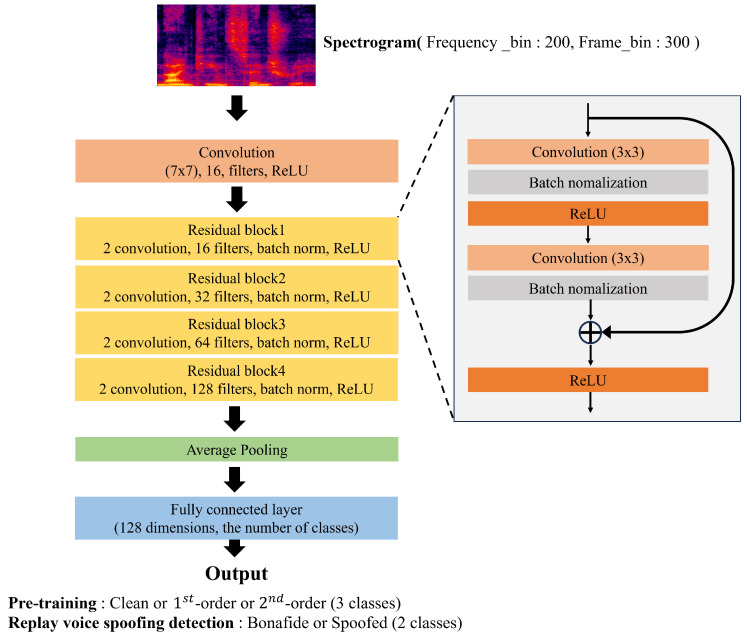
Architecture of pre-training and replay voice spoofing detection model.

Figure 3 shows the architecture of the deep learning model for pre-training and replay voice spoofing detection. The models for pre-training and replay voice spoofing detection have the same Resnet34 [27] architecture, and we performed down-sampling of a number of filters in the convolution layer of the existing model from [64, 128, 256, 512] to [16, 32, 64, 128] for faster convergence of the model. In addition, the updating layer is classified into six layers: convolution, residual block 1, residual block 2, residual block 3, residual block 4, and fully connected layer. The fine-tuning process is performed according to the extent, to which the layer has to be frozen and updated. During training, all the models used the Adam optimizer [28] and cross entropy loss function, with a batch size of 64 and learning rate of 0.001. The number of epochs was 100 for the pre-training model and 30 for the replay voice spoofing detection model, and the learning rate was reduced by a factor of 0.9 every 10 epochs for both the models. We did not use any data augmentation techniques to train the replay voice spoofing detection model.

## 4. Experimental Setup

In this study, we utilized the VCTK Corpus, Aachen impulse response, and ASVspoof2019 PA datasets. The VCTK Corpus dataset consists of utterances and texts from 109 English speakers and provides various versions of the dataset according to loudness, pitch, and timbre. The VCTK Corpus dataset used in this experiment consists of 88,258 English utterances recorded in a hemi-anechoic chamber at the University of Edinburgh using two microphones, the DPA 4035 and MKH 800, with approximately 400 utterances per speaker. The Aachen impulse response dataset is a dataset of RIRs from seven different indoor environments, including offices, kitchens, stairways, and lecture rooms, obtained using a smartphone, totaling 214 RIRs. The ASVspoof2019 PA dataset consisted of replay audios acquired through microphones and speakers such as desktop speakers, Bluetooth device, etc., as well as smartphones. The ASVspoof2019 PA dataset is composed of training data consisting of 5400 original audios and 48,600 replay audios, totaling 54,000 utterances, and an evaluation dataset consisting of 18,090 original audios and 116,640 replay audios, totaling 134,730 utterances. The VCTK Corpus and Aachen impulse response datasets were used for multi-order acoustic simulation to generate the audio that simulated the acoustic configuration of the 1st-order and 2nd-order audio, and thje ASVspoof2019 PA dataset was used to detect the replay audio. In addition, we performed a down-sampling sampling rate of the VCTK Corpus and Aachen impulse response datasets because they have a sampling rate of 48 kHz and 24 bit, while the ASVspoof2019 PA dataset has a sampling rate of 16 kHz and 16 bit. Therefore, we performed down-sampling under identical conditions. For feature extraction, we used a log-spectrogram with magnitude units following a linear scale. We also performed zero padding if the audio was shorter than 3 seconds and sliced it if it was longer. For log-spectrogram extraction, we performed short time Fourier transform with the Hamming window function with a window size of 1024 and hop length of 256 [29].

## 5. Result

Accuracy and F1-score are evaluation metrics that provide an objective measure of the extent, to which a model’s predictions match the actual label in a classification problem in deep learning and are used to evaluate the performance of pre-training models and replay voice spoofing detection models.

**Figure 4:**
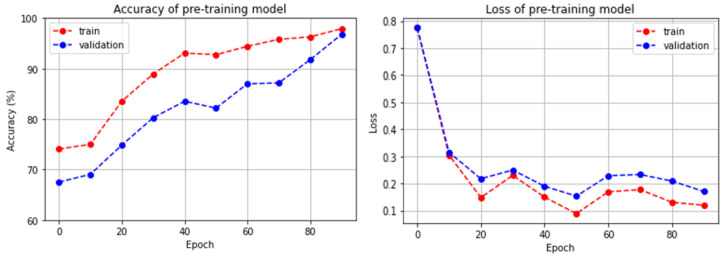
(**Left**) Accuracy of pre-training model on training and validation dataset, (**right**) losses of pre-training model on training and validation dataset.

Table 1 shows the validation dataset generated by the multi-order acoustic simulation to evaluate the performance of the pre-training model. Figure 4 shows the accuracy and loss of the pre-training model on the training and validation datasets per epoch. To generate the validation dataset, the Aachen impulse response dataset was randomized and divided into 150 and 64 RIRs for training and validation, respectively. When training the pre-training model using a multi-order acoustic simulation, we generated the clean, 1st-order, and 2nd-order audios with equal probabilities in mini-batches for the data augmentation effect. However, for the validation dataset, we performed a multi-order acoustic simulation on all the utterances in the VCTK Corpus dataset before training and, finally, generated a validation dataset consisting of 88,258 utterances with 29,365 clean signals, 29,339 1st-order, and 29,554 2nd-order audios. The validation dataset was used to evaluate the performance of the pre-training model, and when fine-tuning for replay voice spoofing detection, we used the weights from the point in the pre-training process that had the highest accuracy on the validation dataset. Table 2 shows that the accuracy and F1-score of the pre-training model on the validation dataset was 98.76% and 96.12%.

Table 3 lists the performance of the replay voice spoofing detection model after fine-tuning the weights of the pre-training model. The method proposed in this study sets the weights of the pre-trained model that classifies the clean, 1st-order, and 2nd-order audios as the initial weights for the replay voice spoofing detection model and performs fine-tuning. The pre-training model and replay voice spoofing detection model used the same Resnet34 architecture, and the layer to be updated during fine-tuning was classified into six layers: convolution, residual block 1, residual block 2, residual block 3, residual block 4, and fully connected layer to evaluate the fine-tuning results according to the layer to be updated. To validate the effectiveness of the proposed method, we compared the performance of the proposed method with that of a conventional method that did not use fine-tuning. The conventional method was trained with the same hyperparameters as the proposed method, and it predicted whether the input audio is bonafide or spoofed through the same Resnet34 architecture using only the ASVspoof2019 PA dataset. Furthermore, we compare the performance with conventional machine learning and deep learning-based methods. The accuracy of the model using the conventional method was 92.94%. When fine-tuning was performed using the weights of the pre-training model, the accuracy was 88.6% when freezing all the weights of resnet34 and updating only the last fully connected layer. However, when updating with residual block4, the accuracy was 93.7%, which is 0.76% better than that of the conventional method. Furthermore, the more layers of the model are updated, the higher the accuracy, which was 96.2% when updating three layers up to block 3, 97.08% when updating two layers up to block 2, and 98.16% and 98.15% when updating block 1 and all the layers. The model with pre-training using a multi-order acoustic simulation showed up to 5.22% higher performance than that of the model without pre-training, and the proposed method showed superior performance.

Table 4 shows the performance comparison between the proposed method and conventional machine learning and deep learning-based replay voice spoofing detection methods. The conventional method using the quadratic filter-based SVM in [30] showed higher performance with an accuracy of 0.64 and F1-score of 3.42 than the proposed method. However, the proposed method outperformed the linear filter-based SVM in [30] with an accuracy of 5.06 and F1-score of 1.85, an accuracy of 16.54 and F1-score 26.58 higher than [31], an accuracy of 1.16 and F1-score of 4.03 higher than [32]. Through experiments, we demonstrated the performance of a multi-order acoustic simulation-based pre-training framework for replay voice spoofing detection.

## 6. Conclusions

In this study, we propose a replay voice spoofing detection method using multi-order acoustic simulation-based pre-training to overcome the limitations of the dataset owing to the physical recording process of the replay. We utilized the VCTK Corpus and Aachen impulse response datasets for multi-order acoustic simulation and ASVspoof2019 PA dataset for replay voice spoofing detection. We assumed that a deep learning model trained on audio simulating different acoustic configurations of 1st-order and 2nd-order audios would be able to classify the different acoustic configurations of the original and replayed audio well. To validate this, we performed pre-training to classify the three classes: clean, 1st-order, and 2nd-order. The weights of the pre-training model were set to the initial weights when training the replay voice spoofing detection model and then performed fine-tuning. To demonstrate the performance of the proposed method, we compared its performance with and without the weights of the pre-training model. The proposed method showed a performance improvement of 5.22% compared to the without pre-training method. We expect that the proposed method will show a higher performance if it utilizes more clean signals and RIR datasets.

## Figures and Tables

**Table 1:** Validation dataset to evaluate pre-training model.

Dataset	Type	Dataset for Pre-Training Model Validation		
		Clean Signal	1st-Order	2nd-Order
VCTK Aachen	Validation	29,365	29,339	29,554

**Table 2:** Performance of pre-training model on validation dataset.

Type	Model	Accuracy (%)	F1-Score (%)
Pre-training	Resnet34	98.76	96.12

**Table 3:** Performance of replay voice spoofing detection models with fine-tuning.

Dataset	System	With Pre-Training	Fine-Tuning Layer	Accuracy (%)	F1-Score (%)
ASVspoof2019 PA	Conventional	-	All layers	92.94	86.92
	Proposed	🗸	FC	88.6	85.58
		🗸	Block 4 + FC	93.7	92.67
		🗸	Block 3, 4 + FC	96.2	93.97
		🗸	Block 2, 3, 4 + FC	97.08	94.12
		🗸	Block 1, 2, 3, 4 + FC	98.16	95.08
		🗸	All layers	98.15	95.12

**Table 4:** Performance comparison with conventional method for replay voice spoofing detection.

System	Accuracy (%)	F1-Score (%)
Banaras et al. [31] (GLGTCC-SVM)	81.62	68.50
lbrar et al. [32] (MLTP-Bi-LSTM)	97	91.05
Javed et al. [30] (ATP + GTCC-SVM (Linear))	93.1	93.23
Javed et al. [30] (ATP + GTCC-SVM (Quadratic))	98.8	98.5
Proposed method	98.16	95.08

## Data Availability

Not applicable.
